# Biogenic Phytochemicals Modulating Obesity: From Molecular Mechanism to Preventive and Therapeutic Approaches

**DOI:** 10.1155/2022/6852276

**Published:** 2022-03-27

**Authors:** Vikram Kumar, Desh Deepak Singh, Sudarshan Singh Lakhawat, Nusrath Yasmeen, Aishwarya Pandey, Rajeev K. Singla

**Affiliations:** ^1^Amity Institute of Biotechnology, Amity University Rajasthan, Jaipur 303002, Rajasthan, India; ^2^INRS, Eau Terre Environnement Research Centre, Québec, QC, Canada; ^3^Institutes for Systems Genetics, Frontiers Science Center for Disease-Related Molecular Network, West China Hospital, Sichuan University, Chengdu 610041, Sichuan, China; ^4^iGlobal Research and Publishing Foundation, New Delhi, India

## Abstract

The incidence of obesity and over bodyweight is emerging as a major health concern. Obesity is a complex metabolic disease with multiple pathophysiological clinical conditions as comorbidities are associated with obesity such as diabetes, hypertension, cardiovascular disorders, sleep apnea, osteoarthritis, some cancers, and inflammation-based clinical conditions. In obese individuals, adipocyte cells increased the expression of leptin, angiotensin, adipocytokines, plasminogen activators, and C-reactive protein. Currently, options for treatment and lifestyle behaviors interventions are limited, and keeping a healthy lifestyle is challenging. Various types of phytochemicals have been investigated for antiobesity potential. Here, we discuss pathophysiology and signaling pathways in obesity, epigenetic regulations, regulatory mechanism, functional ingredients in natural antiobesity products, and therapeutic application of phytochemicals in obesity.

## 1. Introduction

Globally, according to the World Health Organization (WHO), obesity is a major metabolic and heritable disorder, in which more than 1.9 billion adults are suffering from overweight and more than 600 million of them are having the issues of being clinically obese [1, 2]. It is one of the most severe metabolic disorder conditions, which generally develop due to improper energy intake versus energy workout [3–7], stress [8], sedentary lifestyle [9], alcohol consumption [10], depression [11], insufficient nutritional knowledge about the food supplements, and then, ultimately these leads to the accumulation of excessive fats in adipose tissues, which is one of the most prominent factors behind the various chronic disorders such as hypertension, hyperlipidemia, type 2 diabetes, coronary heart disease, and many others [2]. Primarily obesity is an endocrine disorder [12], and interestingly, a recent study also reveals that obesity is not caused by overeating [13].

**Figure 1:**
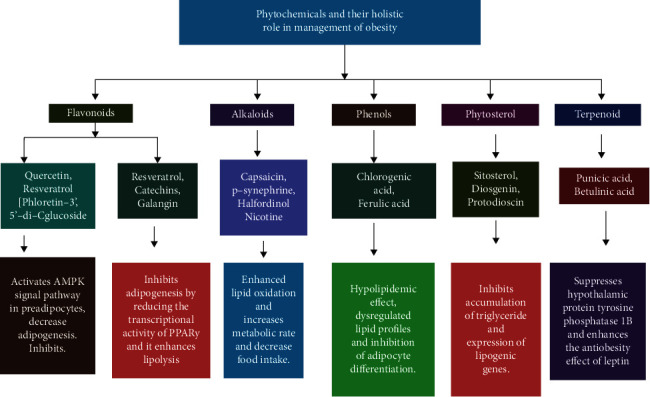
Phytochemicals and their role in obesity to preventive and therapeutic approaches.

Food field research that has recently aroused considerable interest is the potential of natural products to counteract obesity [2, 14, 15]. Nature represents an enormous reservoir of biologically active compounds to treat various ailments from times immemorial [16]. Because of the side effects encountered with long time usage of synthetic drugs and due to stringent guidelines to be fulfilled during drug approvals, plant-herbal drugs have gained much attention as a reliable option to clinical remedy, and the claim for these herbal remedies has greatly increased recently. A variety of phytochemicals are investigated such as polyphenols, alkaloids, terpenoids, flavonoids, tannins, saponins, glycosides, steroids, and proteins present in plants, and their products are key factors in the treatment of several disorders [2]. These products contain dietary phytochemicals with a high potential for health promotion and disease prevention [17, 18]. Multiple phytochemicals combinations may result in synergistic activity that increases their bioavailability and their action on multiple molecular targets, thus offering advantages over treatments with single chemicals [15, 19]. The antiobesity effects of these compounds are mediated by the regulation of various pathways, including lipid absorption, intake, and expenditure of energy, increasing lipolysis, and decrease lipogenesis, and differentiation and proliferation of preadipocytes as shown in Figure 1 [15]. A good number of phytoconstituents such as guggulsterone, hydroxycitric acid, apigenin, genistein, gymnemic acid, caffeine, theophylline, ephedrine, capsaicin, piperine, ellagic acid, and catechins have been reported to possess antilipidaemic and prohealth properties [15, 20, 21]. Although some of these compounds are used in preparing antiobesity drugs/formulations, they lack adequate clinical investigations and scientific validation to be authentic and recommended for obesity therapy.

In other words, the potential of plants, herbs, and their derivatives for the treatment of obesity is still largely unexplored and can be an excellent alternative to develop safe and effective natural product-based antiobesity drugs [22]. The potential of phytochemicals as a source of new drugs opens a wide field for scientific investigation owing to the abundant availability of (250,000–500,000) known species, of which only a small percentage has been phytochemically investigated and evaluated for pharmacological potential [23]. Even from the plants known for traditional medicinal use, many still have not been studied for their effectiveness and safety. Therefore, a necessity has arisen for alternative therapies, especially based on natural products with minimal or no side effects in place of the present therapeutics.

## 2. Pathophysiology and Signaling Pathways in Obesity

**Figure 2:**
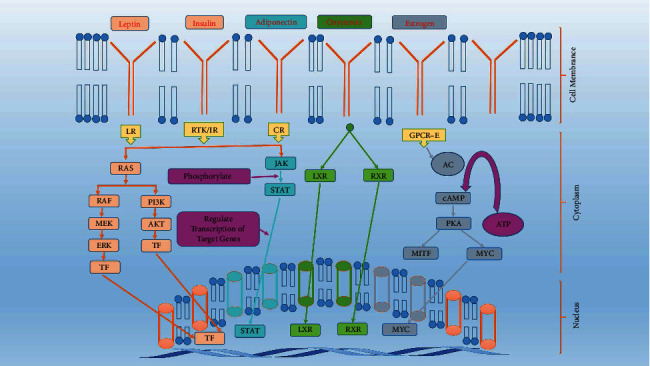
Pathophysiology and signaling pathways in obesity, illustrating different endocrine signaling interactions affected in the progression of obesity. The leptin, insulin, and adiponectin bind to LR, IR, and CR, respectively, and activate different TFs via PI3K and STAT pathways. The estrogen activates cAMP signaling via GPCR-E receptor while oxysterol bind with cell receptors to induce activation of signaling cascade and activate through LXR and RXR transcription factors. The activated transcription factors translocate to the nucleus and bind with DNA to regulate thousands of genes and associated gene expressions. LR: eptin receptor; IR: insulin receptor; CR: cytokines receptor; GPCR-E: G-protein coupled receptor for estrogen; RTK: receptor tyrosine kinase; AC: adenyl cyclase; cAMP: cyclic adenosine 3′,5′-monophosphate; TF: transcription factor; PKA: protein kinase A; MYC: myc proto-oncogene; MITF: microphthalmia-associated transcription factor; RAF: RAF family of serine/threonine kinases; ERK: extracellular-signal-regulated kinase; MEK: mitogen-activated protein kinase kinase; PI3K: phosphoinositide-3-kinase; JAK: Janus kinase; STAT: signal transducer and activator of transcription; AKT: protein kinase B; LXR: liver X receptor; RXR: retinoid X receptor.).

The pathophysiology of obesity is contributed by different endocrine factors and one of the most studied is leptin hormone deficiency. The leptin deficiency triggers hyperphagia mediated through the arcuate nucleus of the hypothalamus. This region expresses neurocircuits involved in the control of feeding process which is regulated by complicated signaling pathways. The variation in nutrition during the gestation period is strongly associated with the onset of neonatal and postnatal obesity. The thrifty gene hypothesis and predation release are two opposite theories that link the evolution process as a cause of obesity. The genome and epigenome studies have associated different genes with obesity. The hypomethylation and hypermethylation of genes and transcriptional factors have been linked with the pathogenesis of obesity. The endocrine-disrupting chemicals (EDC) induce obesity by interfering with various transcriptional factors which regulate fatty acid metabolism, fat absorption, adipocyte differentiation, and adipogenesis as discussed in Figure 2.

### 2.1. Endocrinology of Obesity

The free fatty acids (FFA) are long chains of hydrocarbons with terminal carboxylic acid which bind to glycerol via the esterification process to form fats. The excess amount of energy is stored in form of fat in adipocytes of adipose tissues. During energy-deprived conditions such as fasting, adipocytes release FFA and glycerol which are oxidized aerobically to form ATP [24]. The adipose tissue can be classified as brown adipose tissue (BAT) which is good fat and white adipose tissue (WAT) which is bad fat. This attribute is because BAT is highly vascularised, rich in mitochondria, and is associated with thermogenesis. WAT is most abundant, low in mitochondria, and is least vascularised [25]. The intercapsular BAT is utilized for thermoregulation apart from shivering and sweating to control the body temperature. The adipose tissue not responds to endocrine signals as well as it is involved in the generation of endocrine signals such as leptin and associated cytokines.

The leptin hormone is secreted by adipocytes and is an important link between obesity and homeostasis of energy as shown in Figure 2. The leptin deficiency causes less expenditure of energy and is associated with obesity but exceptionally in many individuals, who are obese, elevated leptin concentrations are found. This probably indicates the prevalence of leptin resistance behind the pathogenesis of obesity [26]. The full effect of leptin in regulating obesity is governed by the central nervous system and particularly important is the hypothalamus.

The hypothalamus consists of agouti-related protein (AgRP) neurons in the arcuate nucleus region. These neurons produce endocrine signals such as neuropeptide Y (NPY), AgRP protein, and gamma-aminobutyric acid which is an inhibitory neurotransmitter [27]. These neurons get activated during fasting due to the reduction of leptin and insulin hormone which induce hunger. The high concentration of leptin and insulin inhibits AgRP neurons. Abnormal activation of AgRP neurons causes hyperphagic, i.e., excessive feeding behavior. Clustered near AgRP neurons, there are Proopiomelanocortin (POMC) neurons in the arcuate nucleus which release *α*- melanocortical stimulating hormone. The activation of these neurons induces satiety [28]. In the deficiency of leptin hormone, POMC neurons are inhibited while AgRP neurons are activated, therefore causing uncontrollable feeding and still induces the feeling of hunger. The genetic mutations in the melanocortin system hence cause hyperphagic obesity. Exceptionally, the calcitonin gene-related protein (CGRP) neurons located in the para branchial nucleus surrounding the superior cerebellar peduncle cause anorexia which is potentially fatal. CGRP-PBN neurons are inhibited by AgRP neurons which increases the meal volume [29].

Since the concentration of leptin is directly proportional to the body fat volume, therefore, generally low leptin concentration is unable to fully activate neurocircuits involved in feedback inhibition of the feeding process and causes obesity [30]. Insulin deficiency or insulin resistance also contributes to obesity by lowering the levels of phospholipase enzyme while the excess phospholipase D1 and D2 expressed in rodents suppress obesity [31]. The postprandial concentration of glucose is regulated by the release of insulin from the pancreatic beta islet of Langerhans which also activates phospholipases [32, 33]. Obesity is a major risk factor for the development of insulin resistance and progression to diabetes which may cause hyperglycemia and leads to cardiovascular disease, sleep apnea, and renal failure [34]. The transition of obesity to diabetes is complex and involves signaling pathways such as Janus kinases (JAK)/signal transducers and activators of transcription factors (STAT) pathway. The binding of growth factor/cytokine on plasma membrane receptors of adipocytes causes stimulation of receptor-associated JKA. The JKA are of 3 types JAK1, JAK2, and JAK3. The signal transducers and transcription factors proteins are of seven types STAT1, STAT2, STAT3, STAT4, STAT5a, STAT5b, and STAT6 containing tyrosine residues that are phosphorylated during activation [22]. The phosphorylated receptor subunit contains specific binding sites for the SH2 (Src homology 2) domain of STAT proteins. After binding to the cytoplasmic tail of JAK proteins in adipocytes, the STAT proteins are phosphorylated at the carboxyl terminus of tyrosine residues. This causes a conformational change in STAT dimer units further to dissociate from JAK and promote their transport to the nucleus. Upon binding with DNA, the STAT proteins activate thousands of genes [35, 36]. The feedback inhibition of this important cytokine signaling involves the suppression of cytokine signaling (SOCS) molecules and protein tyrosine phosphatases (PTP) as shown in Figure 2.

The leptin binds to the beta isoform of leptin receptors, causes activation of JAK-2 by autophosphorylation which further carries phosphorylation of its cytoplasmic tail at tyrosine (Tyr or Y) residues, i.e., Tyr-985, Tyr-1077, and Tyr-1138. This initiates phosphorylation of STAT-3 and STAT-5. The phosphorylation of STAT proteins causes alteration in their conformation and their accumulation in the nucleus to regulate gene transcription [37]. The STAT-3 activation increases gene expression of proopiomelanocortin (POMC) and inhibits AgRP/NPY expression in the arcuate nucleus region of the hypothalamus, thus inducing satiety and inhibiting hyperphagic behavior. The STAT-3 activation parallelly induces the SOCS-3 feedback inhibition loop for the leptin signaling pathway [38]. The protein tyrosine phosphatase (PTP) performs dephosphorylation of JAK and inhibits the JAK/STAT pathway as shown in Figure 2. Experimentally targeted STAT locus deletion in mice causes severe obesity due to probable induction of leptin resistance. The importance of leptin is also indicated by early mutation studies of the ob/ob gene and ob/db gene in mice. The ob gene encodes leptin hormone and the db gene encodes leptin receptor. It is observed that mutations in the ob/db gene reduce responsiveness to leptin presence and may contribute to leptin resistance [39] in individuals. Thus, the absence of leptin in individuals with sufficient energy due to inborn error promotes obesity. The leptin acts on two types of neurons: (a) orexigenic or feed-inducing neurons which expresses NPY and AgRP and (b) anorexigenic or feed-suppressing neurons which expresses cocaine- and amphetamine-related transcript (CART) and *α*-melanocyte stimulating hormone (*α*-MSH). In the arcuate nucleus, leptin-regulated proopiomelanocortin (POMC) is an important precursor for the *α*-MSH hormone which binds to the melanocortin-4 receptor (MC4R) and induces anorexic and feeding suppression properties. The AgRP and *α*-MSH are antagonistic for the MC4R receptor and produce opposite effects upon binding [40]. The MC4R receptor is activated by *α*-MSH and hence suppresses feeding behavior. MC4R inhibition by AgRP promotes feeding and decreases leptin response. Similar to AgRP, the NPY also increases the feeding process while suppresses catabolism and promotes anabolism [41]. The insulin suppresses both food intake and expression of NPY in the arcuate nucleus. The study of agouti mice revealed that the agouti protein mimics the structural properties of *α*-MSH, and therefore, it inhibits *α*-MSH binding to MC4R receptors. A similar heterozygous mutation at this locus reported in four to five percent of human beings causes severe obesity. The regulation of paraventricular hypothalamic nuclei by leptin-controlled arcuate nucleus neuron terminals is strongly involved in the pathogenesis of obesity.

### 2.2. Neonatal Development

The intake of food is regulated by stomach distention in infants. In neonates, the arcuate nucleus projects into the brain and expresses the POMC gene. During development, arcuate nucleus then sequentially express NPY followed by GABA and then AgRP [42].

Random axonal growth occurs from the first to the third week of the postnatal period. During the gestation period, depending upon the metabolic state, the signals from mother to fetus influences the property of AN neurons to increase the volume of POMC neurons or NPY forms, i.e., anorexigenic or orexigenic forms. Both overnourishment as well as undernourishment of mother during pregnancy is associated with a risk factor of obesity in the offspring. Incidentally, maternal obesity during pregnancy reduces the mass of neurons that expresses leptin receptors and may cause leptin resistance, while undernutrition or malnutrition causes the delayed response to presynaptic GABA and postsynaptic AgRP neurons to stimulus [42, 43].

The leptin deficiency is also associated with decreases in BAT stores. The most accepted yet controversial hypothesis of thrifty genes in human beings states that obese individuals are evolutionarily superior to survive in starving conditions such as famine or flood. Hence, it is the normal approach of the body to defend against stressful conditions by storing more energy in form of fats. This energy in obese individuals may be used in adverse conditions [44]. In opposition to the thrifty gene hypothesis, predation release theory hypothesizes that societal development and invent of weapons by humans have resulted in superiority over much stronger predators of the ancient era which removed the pressure of natural selection and survival of the fittest against predators. This resulted in the drifting of obesity traits overcoming generations [45]. The genome-wide association study (GWAS) is a new technique used to associate specific genotypes with diseased phenotypes. The method involves analysis of the genome in masses and then identification of genetic markers to envisage the disease. It has recognized different gene variants for obesity. The GWAS analysis in mice has associated gene mutations in Apoe, Ppm1l, Lpl, and Lactb genes with obesity. These mutations cause defective apolipoprotein E, protein phosphatase 1 like protein, lipoprotein lipase, and lactamase b, respectively, that are strongly associated with obesity [46]. Some of the literature also indicates that genetic engineering of wheat and other cereal crops is responsible for causing obesity. It is predicted that the genetic engineering of wheat varieties has caused a modification in starch content and therefore induce obesity [47]. The interaction between genes and environment and particularly the sedentary lifestyle along with elevated stress levels also are associated with obesity. Fat mass and obesity (FTO) associated gene is expressed in adipocytes and hypothalamus which encodes FTO protein (alpha-ketoglutarate dependent dioxygenase). A single base pair variation within the first intronic sequence of FTO proteins is associated with obesity in individuals [48]. This means that not only the disruption of coding sequences of DNA but also noncoding sequences play a major role in obesity.

### 2.3. Epigenetic Modifications

Epigenetics is the study of heritable phenotypic traits caused by modifications in chromosomes without significant variations in sequences of DNA. The epigenome-wide association analysis (EWAS) has associated hypermethylation and hypomethylation of DNA in WAT with proinflammatory pathways and obesity. The hypermethylation of *β*-adrenoceptor (ADRB3) and hypomethylation in the leptin (Lep) gene are associated with obesity. The folic acid deficiency also suppresses methylation by inhibiting the DNA methylase enzyme. Endocrine-disrupting chemicals (EDCs) induce hypermethylation of DNA sequences and promote obesity [49]. EDCs are environmental chemicals that interfere with the endocrine system and promote obesity. Some of the EDCs are tributyltin, diethylstilbesterol, dichlorodiphenyltrichloroethane (DDT), phytoestrogens, parabens, etc. The transcription factors such as peroxisome proliferator-activated receptors (PPARs) are classified into PPAR gamma, alpha, and epsilon, which regulate and control genome throughout the life wherein EDCs binding with nuclear receptors of PPAR gamma is related to obesity. The PPARs are ligand-activated transcription factors that cause the formation of the dimer with retinoid X receptors (RXRs) and promote adipocyte differentiation and adipogenesis. Maternal obesity as well as maternal malnutrition both are associated with risk factors of obesity in the offspring. During gestation, maternal obesity is associated with a high risk of being transferred to offspring caused by decreased methylation of developmental gene Znf483. Likewise, early gestation exposure to malnutrition increases risk factors of obesity [50–52]. The gut microflora dysbiosis is also the most important contributor to obesity. The bacterial strains of Gram-positive Firmicutes and Gram-negative Bacteroidetes form general gut microflora. The higher ratio of Firmicutes : Bacteroidetes is associated with the risk of obesity [53].

### 2.4. Metabolism of Fatty Acids

During starvation and low glucose levels, the adipose tissue releases FFA in blood which are then utilized as an energy source by starving cells and tissues. The fat synthesis in the body occurs in two ways via ingestion of a fat-rich diet and by biosynthesis of fat [54]. Upon ingestion, the fat undergoes lipolysis by forming FFA and glycerol by intestinal lipases. They are then transported to intestinal enterocytes cells through transporter protein. Once inside the enterocyte cells, the FFA and glycerol are reassembled to form triglycerides [55]. At the same time, the cholesterol is converted to cholesterol esters. Finally, the association of triglycerides, cholesterol, and lipoprotein in form of chylomicrons is completed. The very low-density lipoprotein and triglycerides are produced in the liver. Both chylomicron and very-low-density lipoprotein under the stimulation of insulin hormone and lipoprotein lipase enzyme transports FFA and triglycerides into muscles and adipose tissues via the blood. The residual chylomicrons and very-low-density lipoprotein decrease in size and then forms low-density lipoprotein. They are then consumed by hepatocytes which lower cholesterol levels and low-density lipoprotein levels. The HDL or high-density lipoprotein removes cholesterol from extrahepatic peripheral tissues and transports it to the liver [56]. During fat biosynthesis, the glucose under energy-rich conditions is converted into fatty acids via the formation of malonyl coenzyme A and involving fatty acid synthase complex [57]. The adipocytes hypertrophy is a condition in which there is an increase in the size of the adipocytes which leads to obesity while adipocyte hyperplasia is a condition in which there is an increase in numbers of adipocytes caused by differentiation of preadipocytes which also leads to obesity. Mild obesity involves adipocyte hypertrophy Severe obesity involves both hypertrophy and hyper plasticity [58]. Hyperplastic adipocytes are metabolically active and generally harmless but hypertrophic adipocytes are pathogenic and store unhealthy fat. Due to hypoxia and low space, they produce inflammatory cytokines such as interleukin-6, tumor necrosis factor-alpha, and leptin but downregulate production of adiponectin hormone [59]. Adiponectin is an anti-inflammatory hormone that is released by adipocytes. It is important in the regulation of the sugar level and fatty acid breakdown [60]. At the same time, obesity and overweight are the main carters of metabolic syndromes and nonalcoholic fatty liver disease (NAFLD).

NAFLD is a continuum of hepatic ailments linked with metabolic and cardiovascular disorders, such as obesity, dyslipidemia, type 2 diabetes, insulin resistance, and hypertension. It is characterised by increase in liver fat contents (>5%) followed by inflammation and fibrosis [61, 62]. In this condition, the FFA release rate from adipose tissue and delivery to skeletal muscle and liver is also increased in obese subjects with NAFLD, which outcomes in escalation in muscle and hepatic FFA uptake. Other than this, intrahepatic de novo lipogenesis (DNL) of FFA is more in subjects suffered with NAFLD as compared with normal intrahepatic triglyceride (IHTG), which promote the accretion of intracellular fatty acids [61]. The formation and release of TG in VLDL is amplified in subjects with NAFLD, which provides a mechanism for removing IHTG. Augmented blood sugar and insulin accompanying with NAFLD stimulate DNL and slow down the fatty acid oxidation, by distressing sterol regulatory element binding proteins (SREBP-1c) and carbohydrate responsive element binding proteins (ChREBP). All these abnormal metabolic progression proliferate the intracellular fatty acids that are not oxidized or disseminated within VLDL-TG, and which are subsequently esterified to TG and deposited within lipid vesicles and at the same time, some lipid metabolites of fatty acid can impair the insulin signaling process which leads to the tissue insulin resistance [61, 62].

### 2.5. Adipogenesis

The preadipocytes convert into mature adipocytes via two processes: preadipocyte proliferation and adipocyte differentiation. The adipocyte differentiation involves sequential morphological changes: (a) growth arrest, (b) mitotic clonal expansion, (c) early differentiation, and (d) terminal differentiation. All the above steps are triggered by preadipocyte factor 1 (pref-1) and CCAAT enhancer-binding protein (C/EBP) [63, 64]. The C/EBP-beta and -delta are transcription factors that induce activity and expression of peroxisome proliferator-activated receptor-gamma (PPAR-gamma). The PPAR-gamma is an inducer of C/EBP-alpha that binds to the promoter of PPAR-gamma and -alpha and promotes the differentiation process [64]. After completion of the differentiation process, all markers of mature adipocytes are well expressed. To produce energy, the triglycerides degrade into FFA and glycerol in absence of glucose as a carbon source. There is a natural hierarchy for catabolic activities to generate energy. In this hierarchy, carbohydrates reserves are first consumed to generate energy followed by lipids and then the proteins [65]. The WAT performs the breakdown of triglycerides into FFA and glycerol during lipolysis. In the cytoplasm of WAT, the adipose triglyceride lipase, monoacylglycerol lipase, and hormone-sensitive lipases in acute energy need to convert triglycerides into FFA and glycerol. These FFA are used as energy sources in organs and tissues via beta-oxidation [66]. The energy imbalance within the adipocytes produces endoplasmic reticulum stress and mitochondrial stress. The free fatty acid oxidation occurs in two sites namely peroxisomes and mitochondria. The free fatty acid oxidation in peroxisomes is strictly utilized for free fatty acid biosynthesis; on the other hand, mitochondrial free fatty acid oxidation is used for energy production during the crisis. The obese individual tends to depend upon glucose oxidation more than fatty acid oxidation revealing that mitochondrial fatty acid oxidation is compromised [67].

## 3. Progression of Antiobesity Pharmaceuticals

Obesity is now a global problem [68] and is associated with several chronic conditions including osteoarthritis, obstructive sleep apnea, gallstones, fatty liver disease, reproductive and gastrointestinal cancers, dyslipidemia, hypertension, type 2 diabetes, heart failure, coronary artery disease, and stroke [69]. However, many medications have been used to manage obesity over the years. For research scientists, there are now numerous molecular targets for antiobesity drugs: central receptors for biogenic amines, cannabinoids, hypothalamic neuropeptides, peripheral *β*3-adrenoceptor, and UCPs; and dominating all obesity research is leptin—a tantalizing but frustrating obesity target. However, most of the antiobesity drugs that were approved and marketed have now been withdrawn due to serious adverse effects. In the 1990s, fenfluramine and dexfenfluramine were withdrawn from the market because of heart valve damage [70]. In 2000, the European Medicines Agency (EMA) recommended the market withdrawal of several antiobesity drugs, including phentermine, diethylpropion, and mazindol, due to an unfavorable risk-to-benefit ratio [71]. The first selective CB1 receptor blocker, rimonabant, was available in 56 countries from 2006 but was never approved by the U.S. Food and Drug Administration (FDA) due to an increased risk of psychiatric adverse events, including depression, anxiety, and suicidal ideation [72]. Subsequently, rimonabant was withdrawn from the European market in 2009. Recently, many newer agents have been tried, though only orlistat and sibutramine have been approved for long-term use. In October 2010, sibutramine, widely used after approval by the U.S. FDA in 1997, was withdrawn from the market because of an association with increased cardiovascular events and strokes [73]. More recently, in February 2011, the U.S. FDA rejected approval of the bupropion/naltrexone combination marketed as Contrave due to concerns over potential cardiovascular risks. Currently, some of the US-FDA approved antiobesity active pharmaceutical ingredients, i.e., phentermine, lorcaserin, naltrexone, orlistat, and liraglutide are clinically tested (Table 1) and available in the market [74].

The long-term safety and efficacy of newly developed drugs should also be evaluated in the management of obesity, which often requires continuous treatment to achieve and maintain weight loss, though the rigidity of a regulatory committee for the approval of novel antiobesity drugs and the regulatory guidelines for antiobesity therapy represents a significant limitation to developing drugs [75, 76].

Antiobese drugs generally have the potentials to decrease appetite or increase satiety, such as sibutramine; those that are adrenergic and also reduce the appetite, such as the amphetamines; and those that alter the digestion, such as orlistat. Besides, most of the treatments available have only revealed short-term assistances and are not recommended for sustained weight loss due to the liver enzyme abnormalities and liver disease. Fenfluramine is an example of a drug that is no longer approved for the treatment of obesity because of safety concerns. Elevations of aminotransferases and/or alkaline phosphatase have been reported with sibutramine use in placebo-controlled trials [77]. Drugs used for the management of obesity comorbidity disease such as NAFLD, diabetes, hypertension, and hyperlipidemia also have serious hepatotoxicity. Antidiabetic drugs, i.e., glibenclamide/glyburide, chlorpropamide, tolbutamide, tolazamide, metformin, pioglitazone, and rosiglitazone are reported for hepatotoxicity in terms of cholestasis, hepatitis, and liver failure.

Antihypertensive drugs, i.e., nifedipine, diltiazem, amlodipine, losartan, candesartan, valsartan, enalapril, ramipril, captopril, and fosinopril are also reported for severe hepatitis in liver [78]. At the same time, some natural products or phytochemicals are also reported for hepatotoxicity, which have been widely used for the obesity management since last many decades [79]. The extract of *Germander* causes the drug-induced liver injury (DILI), which is probably mediated by furano neoclerodane diterpenoids. The desert shrub *Chaparral* was marketed for weight loss, but its active phytochemical nordihydroguaiaretic acid is reported to have the liver toxicity in recent studies. *Kava* preparation of rhizome of *Piper methysticum* marketed for anxiolytic and mood enhancer, but some studies described the kava associated immune-mediated liver toxicity, with CYP2D6 deficiency. The roots of “*Chelidonium majus*” herb contain the biologically active phytoconstituents chelerythrine and sanguinarine, and their principia are similar to the opium and reported for hepatotoxic effects. Phytochemicals of “*Lycopodium serratum*” also reported for idiosyncratic or hypersensitivity reaction based cholestatic type hepatotoxicity [80]. In view of such serious toxicity of herbal or natural products, there is a great need of authenticated safe and effective treatment for obesity and its comorbidity diseases.

## 4. Obesity Regulating Mechanisms of Natural Phytochemicals

### 4.1. Proliferation in Energy Outflow

**Figure 3:**
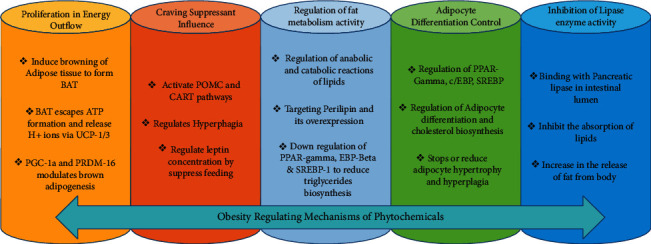
Obesity-regulating mechanisms of natural phytochemicals.

The BAT is highly vascularised, rich in mitochondria, and is strongly associated with heat generation or thermogenesis. During low-temperature conditions, the BAT helps in the regulation of body temperature [81]. The BAT consumes excess energy for heat generation by a process that involves leakage of protons produced during the electron transport chain and oxidative phosphorylation [81, 82]. Interestingly, BAT escapes ATP formation and results in leakage of protons from perimitochondrial space through uncoupling protein (UCP-1 or UCP-3) which generates excess energy in form of metabolic heat and also promotes higher oxidation rates of FFA [82]. The WAT which stores unhealthy fat can be converted into BAT in a process of adipocyte differentiation known as browning. Therefore, the proliferation of BAT and browning of WAT can contribute towards the treatment of obesity by releasing excess stored energy in form of heat [83]. Natural phytochemicals have been found helpful in the treatment of obesity. Particularly, the phytochemicals rutin, naringenin, luteolin, and quercetin promote browning of WAT and prevents obesity (Table 2). The genistein and myricetin cause prevention of obesity by elevation of UCP-1 expression, peroxisome proliferator-activated receptor gamma coactivator 1-alpha (PGC-1a), and PRDM-16 which promotes brown adipogenesis through PPAR-gamma as shown in Figure 3 [84].

### 4.2. Craving Suppressant Influence

The reduction of appetite or induction of satiety can be of great significance in the regulation of obesity. The studies revealed that certain systems in the central nervous system are modulators of metabolic activities. The hypothalamus is one such major modulator in regulating hunger [85]. The uncontrolled hunger in which satiety is not achieved due to inborn errors of metabolism is related to obesity. The arcuate nucleus in the hypothalamus expresses NPY and AgRP which induces hunger and food craving while the CART and POMC pathway suppresses hunger [86]. The recent advances in obesity treatment have considered apigenin flavonoid to regulate hyperphagia or excessive feeding behavior. The apigenin activates POMC and CART pathways in the brain [87]. The other similar phytochemicals include isoflavone genistein and flavonoid cyanidin which also suppresses feeding by regulation of leptin concentration in the brain (Table 2). These approaches shall be greatly helpful in controlling obesity in the future [88, 89] as shown in Figure 3.

### 4.3. Inhibition of Lipase Enzyme Activity

One of the important factors which contribute to obesity is the elevated absorption of lipids and carbohydrates via the gastrointestinal tract. The inhibition of such digestive enzymes which carry absorption of carbohydrate and lipid from the intestine shall reduce obesity [90]. The enzyme phospholipase is secreted in pancreatic juice which triggers the breakdown of triglycerides into FFA and monoglycerides in the intestinal lumen. After absorption of FFA and monoglycerides in blood, they are transported towards chylomicrons for reassembly of the triglycerides and the formation of cholesterol esters [91]. The inhibition of the pancreatic phospholipase enzyme can be promising for the treatment of obesity. The side effects studies of such inhibition processes must be thoroughly performed, since it may cause stomach flatulence and distention. An inhibitor of the alpha-amylase enzyme may prevent fatty acid biosynthesis under excess consumption of glucose and related sugars [92]. Generally, under low energy requirements, excess glucose is converted into fatty acids via the formation of malonyl CoA. In this process, the mitochondrial citrate is transported to the cytoplasm where it is used to regenerate acetyl coenzyme A. The acetyl coenzyme A is converted to malonyl coenzyme A by acetyl-coenzyme A carboxylase enzyme. The cytoplasmic fatty acid synthase complex uses malonyl CoA and synthesizes fatty acid chains [93]. The plant-derived flavonoid luteolin is a strong phospholipase inhibitor. Similarly, epigallocatechin-3,5-digallate and other related flavan-3-ol-digallate esters offer very high phospholipase inhibition properties [94] (Table 2). One of the latest isolated flavone compounds has shown the highest inhibition level alpha-amylase enzyme, as shown in Figure 3 [95].

### 4.4. Adipocyte Differentiation Control

Certain phytochemicals possess adipocyte proliferation and differentiation regulatory properties. The formation of new adipocytes is referred to as adipogenesis. It is regulated by adipocyte-specific genes belonging to PPAR-gamma, C/EBP families such as C/EBP alpha, beta, and epsilon. Interdependence of C/EBP alpha and PPAR-gamma controls adipocyte differentiation process [96]. Sterol regulatory element-binding proteins (SREBPs) also play important role in cholesterol balance, free fatty acid metabolism, and differentiation of adipocytes. The SREBP 1a, SREBP 1c, and SREBP 2 induce cholesterol biosynthesis. SREBP 1c promotes differentiation of adipocytes and may activate PPAR-gamma. The inhibition of C/EBP alpha, PPAR-gamma, and SREBP may be effective for obesity treatment [97]. The phytochemicals such as apigenin [98], guggul sterols [99], naringenin, genistein [100], hesperidin, myricetin, kaempferol, and rutin [101] have been proven effective in downregulating the differentiation of adipocytes (Table 2). This inhibition of adipocyte differentiation by apigenin is linked with possible inhibition of interleukin 6, leptin production, and monocyte chemoattractant protein 1 (MCP-1). The genistein suppresses the expression of PPAR-gamma, SREBP-1c, and GLUT-4 via JNK signaling. Myricetin interacts with PPAR-gamma and decreases adipocyte differentiation, as shown in Figure 3 [102].

### 4.5. Regulation of Fat Metabolism Activity

The release of FFA and glycerol in adipocytes is controlled by several mechanisms. The stores of WAT are determinants for the plasma concentrations of FFA. The elevated rates of lipolysis during feeding process contribute to the high ratio of circulating fatty acid level. Therefore, the phytochemical which may target the important steps in the catabolism of fats shall prevent obesity [101]. Likewise, perilipin lipid droplet coating protein blocks the lipases to reach triglycerides present in adipocytes. The enzymes protein kinase A causes phosphorylation of perilipin and induces conformational change. This promotes the binding of protein lipase A and initiates lipolysis. The low perilipin has been proven helpful for the treatment of obesity in high-fat-diet-fed rats [103]. The genistein along with daidzein downregulated perilipin-1. It also promoted lipolysis and inhibited insulin-dependent lipogenesis [104]. The kaempferol downregulated PPAR-gamma/EBP beta, SREBP-1, and genes of triglyceride biosynthesis [105]. Apigenin, kaempferol, and hesperidin (Table 2) decrease triacylglycerol concentrations in adipocytes by regulating lipolysis, as shown in Figure 3 [106].

## 5. Functional Ingredients in Natural Antiobesity Products

### 5.1. Plant Metabolites/Phytochemicals

The plethora of investigations conducted on plant metabolites provides ample evidence that demonstrates the therapeutic potential of these phytochemicals in treating obesity and the related diseases. Millions of phytochemicals exist that are broadly classified under polyphenols, alkaloids, and terpenoids. These phytochemicals/plant metabolites exert their antiobesity effects (Table 3) either alone or synergistically via the following mechanisms: (a) enhancing energy expenditure, thermogenesis, lipolysis, and lipid metabolism, modulating adipose tissue, acting as appetite suppressants, regulating adipogenesis, inhibiting the activity of the enzyme pancreatic lipase, acting as antioxidants, and preventing oxidative damage in living systems [107].

### 5.2. Polyunsaturated Fatty Acids (PUFAs)

One of the most important ingredients used to combat obesity is the type of dietary fat being used. Although people opine that fat is the main reason for obesity but that does not hold true as the amount and type of fat being used plays a crucial role in weight management via modulating metabolism and adipose tissue function [108]. Consequently, dietary fats rich in monounsaturated fatty acids (MUFAs) and polyunsaturated fatty acids (PUFAs) are considered to enhance body metabolism. PUFAs are known to show excellent antiobesity effects and are further classified into *ω*-3 and *ω*-6 groups. *ω*-3 PUFAs consist of two types of fatty acids, one is eicosapentaenoic acid (EPA) and the other one is docosahexaenoic acid (DHA). There is an intermediate fatty acid between EPA and DHA known as docosapentaenoic acid (DPA) [109]. PUFAs can be obtained from fish and fish oils. Numerous investigational studies reported that dietary fatty acids are involved in modulating adipose tissue properties and homeostasis between glucose and insulin. Substantial evidence from literature quote several mechanisms by which *ω*-3PUFAs could exert their antiobesity effects: (a) increase in fatty acid *β*-oxidation, (b) enhanced energy expenditure via thermogenesis, (c) alteration in epigenetic effects and gene expression in fat tissue, (d) modulation in adipokine pathways leading to altered adipokines release, and (e) reduction in enzymatic activity of fatty acid synthase and stearoyl-CoA desaturase-1 (lipid synthesizing enzymes) finally appetite suppression [110]. However, a balanced *ω*-6/*ω*-3 ratio of 1 : 2/1 along with physical activity of some degree is identified to be a key factor in the treatment of obesity and related diseases [111].

### 5.3. Dietary Fiber

The role of dietary fibers (DFs) in treating obesity and related disorders has become prominent. DFs are either analogous carbohydrates or edible plant parts that exhibited altered digestion patterns in the human intestine. DF is of two types: soluble dietary fiber (SDF) and insoluble dietary fiber (IDF). SDF is generally fermented by the microbiota of the gut releasing short-chain fatty acids (SCFAs) metabolites. SDFs (especially guar gum, pectin, psyllium, and *β*-glucans) are viscous [112]. This physicochemical property of SDFs helps them to form a barrier like gel in the small intestine which enables to delay absorption and gastric emptying altering postprandial metabolism. On the other hand, IDF which is seen in cellulose, hemicellulose, and lignin is regarded as fecal bulk-forming agents [113]. The physiological mechanisms contributing to weight loss strategies include (a) appetite suppression through satiating properties of the fiber, (b) delayed gastric emptying, (c) alteration in the transport mechanism involving glucose and fat, (d) its fiber regulates the defecation rhythm, and (e) it has a role in the growth of gut microbiota [114]. Evidence from studies reported that DF is fermentable by the gut microbiota which in turn helps in the production of SCFAs which are known to enhance satiating properties of DF that is inversely proportional to the weight gain in humans [115]. DF also altered blood lipid concentrations and glycemic response in humans. There exists robust epidemiologic evidence that proves that dietary fiber intake can help overcome overweight and obesity; however, many more interventional studies are needed to ascertain these effects removing all the practical interventional hurdles.

### 5.4. Proteins

A balanced diet essentially consists of dietary proteins. A diet that has 1.2–1.6 gm of protein/kg/day and which has approximately 25–30 gm of the protein-rich meal regarded as higher protein. A high-protein diet (HPD) is generally associated with weight loss strategies, improved satiety, fat mass loss, etc. Evidence from scientific studies has put forth several mechanisms by which dietary protein can help in weight loss and management: (1) improved satiety that is associated with enhanced thermic effects and (2) enhanced energy expenditure [116]. Dietary proteins also enhance the fat-free mass which is attributed to an improvement in body composition. It is evident from research data that HPD elevates levels of anorexigenic hormones such as glucagon-like peptide-1, cholecystokinin, and peptide tyrosine-tyrosine whilst reducing levels of orexigenic hormones (such as ghrelin), ultimately increasing satiety and reduction in food intake. HPD induced thermic effects also called diet-induced thermogenesis (DIT) might be due to increased amino acid oxidation levels postprandial resulting in increased oxygen demand essential for the movement of proteins and finally enhanced satiety. Hence, it can be concluded that HPD can be used as a safe tool for efficacious weight reduction and weight management to overcome obesity and the related diseases. However, to confirm the beneficial effects of HPD, in-depth clinical trials for a longer period must be conducted [117].

### 5.5. Antiobesity Effect of Dietary Calcium

Calcium (Ca) is regarded as the most versatile nutrient that acts as a prolific second messenger in several physiological processes and a signaling agent. Calcium helps in the fertilization and development process, nerve signal transduction, cell proliferation, neuronal plasticity, flexibility in mammals through muscular movements, skeletal integrity, bone metabolism, blood coagulation, cellular differentiation, and cell death [118, 119]. The array of diverse functions exhibited by Ca^2+^ led scientists to investigate its role in diseases. Epidemiologic data from several studies indicate that there exists a strong association between dietary calcium intake and the prevalence of obesity [120]. The plausible mechanisms by which the amount calcium intake can regulate the bodyweight or modulate obesity are as follows: (1) high Ca^2+^ intake increases fecal fat and EE (energy expenditure). This is probably due to increased dietary calcium which binds to more fatty acids and or to bile salts in the colon, leading to the formation of insoluble calcium-fatty acid soaps and thereby inhibiting fat absorption and hence reducing weight [121]. (2) Intracellular calcium levels are regulated by hormones, such as parathyroid hormone (PTH) and 1,25-hydroxyvitamin D (1,25-(OH)(2)-D). High dietary calcium intake could stimulate lipolysis, inhibit fat accumulation mainly by preventing the release of parathyroid hormones and 1,25-(OH)(2)-D. Hence, it is opined that calcium intake can influence lipogenesis or lipolysis based on the influx of Ca^2+^ in adipocytes [122]. Several studies in both humans and animals were conducted but the data reported are inconsistent related to the impact of Ca^2+^ intake on anthropometric measurements and the prevalence of obesity warranting further elaborative investigation with conclusive results.

### 5.6. Probiotics

Microbes that are commensals in the human intestines play a crucial role in the absorption, lysis, and storage of nutrients. They are also essential in several physiological processes of humans such as metabolism, digestion, circadian rhythms, and vitamin synthesis [123]. Additional roles of gut microbiota are to influence tissue-fatty acid composition, stimulate energy production, and inflammation. Gut microbiota dysbiosis is found to be inherently associated with metabolic disorders leading to obesity. Modulation of gut microbiota using nutraceuticals (probiotics and prebiotics) has shown promising effects in the treatment of obesity [124]. Probiotics are nutraceuticals composed of live bacteria, especially Lactobacilli and Bifidobacteria, which help in improving gut microbiome and health status. The gut microbiome along with the probiotic supplementation has a positive impact on health conditions such as type 2 diabetes, immune, infectious diseases, and also cardiovascular diseases. Its impact on the neurotransmission signaling and functionality of the brain has a significant influence on weight management and obesity. Probiotics help in weight loss by inhibiting fat accumulation, leading to inflammation reduction, and also have the capability to reduce insulin resistance. Apart from this, they influence gastrointestinal peptides and neuropeptides [125]. Prebiotics are dietary substrates defined as “dietary ingredients that on selective fermentation lead to changes, gut microbiota especially its composition and function those of which are associated with potential health benefits to the host” [126]. Several studies conducted reported that usage of prebiotics especially insulin-type fructans help in the colonization of arabinoxylan, *Roseburia*, and *Clostridium* cluster XIVa, which further increases species such as *Bacteroides* and *Bifidobacterium* along with *Roseburia* exhibiting the antiadipogenic effect in obese mice fed with the high-fat diet. Although these findings prove the importance of prebiotics and probiotics in obesity management with experimental evidence from animal studies, significant supportive data from human clinical trials are unavailable [127, 128].

## 6. Therapeutic Application of Phytochemicals in Obesity

Some clinically important secondary metabolites are used as antiobesity agents [129]. Several phytochemicals are employed to reduce the process of adipogenesis, carbohydrates absorption in the small intestine, collection of hepatic triglycerides, deposition of adipose tissue, weight loss, and enhances the antiobesity potential, activity of PPAR-*α* and PAR *γ*-responsive genes (Table 3) [130, 131]. Flavonoids have the potential to inhibit lipase activity and control adipogenesis, such as quercetin, catechins, resveratrol, and galangin. Cyanidin and cyanidin 3-glucoside molecules isolated from *Ribes nigrum* and *Morus alba*, respectively, both molecules reduce triglycerides and normalized adipocytokine secretion [131–134]. Adipogenic transcription factor (C/EBP) increases lipolysis and fatty acid oxidation and normalizes adipocytokine secretion. Some other flavonoids and genistein increase the HDL (high-density lipoprotein) and decrease the BMI (body mass index). Naringin and berberine reduce the hypercholesterolemic level, decrease the blood lipid and quercetin molecules, and increase the blood HDL [134, 135]. Alkaloid with antiobesity property isolated, synephrine from *Citrus aurantium*, nuciferine from *Nelumbo nucifera*, piperine and piperlongumine from *Piper nigrum*. Green coffee reduced body fat by decreasing the absorption of glucose [136–138]. Phenols are an important phytochemical and reduce the risk of obesity such as p-hydroxybenzoic, cinnamic acid, ferulic acid, caffeic acid, p-coumaric, and cinnamic acid [139–141]. Phytosterols are naturally occurring phytochemicals and reduce obesity and decrease LDL-cholesterol such as campesterol, brassicasterol, guggulsterone, sitosterol, diosgenin, and stigmasterol and appear to reduce obesity, and high intakes of these compounds decrease LDL-cholesterol levels in the intestinal lumen. Phytosterols compete with cholesterol for micelle [142–144]. Chemically modified terpenes are found in plants and consist of both primary and secondary metabolites [145–148]. Some terpene molecules are ligands with hermeneutic potential to stimulate PPAR and act as dietary sensors and regulate glucose metabolism and control energy homeostasis metabolism [145–148]. In the recent era of research and development, the natural phytochemicals are also playing very important role for the treatment of NAFLD. Epidemiological evidence recommends that a healthy dietary habits with cumulative intake of several plant-based natural phytochemicals, i.e., spirulina, berberine, oleuropein, garlic, curcumin, resveratrol, coffee, ginseng, glycyrrhizin, cocoa powder, bromelain, and epigallocatechin-3-gallate could lower the risk of NAFLD [149, 150], and researchers are working to develop the phytochemicals based on conventional, safe, and effective dose formulations via the clinical studies. Some of the important bioactive components of antiobesity preclinical screening details are discussed in Table 4.

## 7. Clinical Studies: Translational Potential of Natural Products from Bench to Bedside

There are very limited clinical studies reported on the antiobesogenic activity of numerous herbal plant extracts and their active phytochemicals. Hydroxyl citric acid isolated from the herbal plant *Garcinia cambogia* has been reported to have potent lipogenesis and ATP citrate lyase inhibitor activity and decreased serum triglyceride in obese women [162]. Isolated natural phytoconstituent curcumin from *Curcuma longa*, polyphenols from *Salacia oblonga*, terpenoids from *Emblica officinalis* exposed to have hypolipidemic, blood glucose-lowering, and antioxidant activity in diabetic and obese patients during the clinical studies [163]. Herbal tea extract of *Salacia oblonga* has the potential to improve the lipid profile and decrease fasting blood glucose and HbA1c levels in obese and diabetic patients [164]. Crude extract of *Portulaca oleracea* L. seeds also has a significant role in ameliorating lipid profiles in obese adolescents [165]. *Hibiscus sabdariffa* plant extract is stated to decrease the body weight, BMI, body fat, and waist-to-hip ratio in clinical studies with a BMI of ≧27 and aged 18–65 [166]. Dietary supplement with *Phaseolus vulgaris* extracts was also reported to have the *α*-amylase-inhibiting activity, which significantly reduced the body weight, fat mass, and waist/hip perimeters [167]. The antiobesity mechanisms reported for *C. annuum*, in which the effect on fat oxidation was observed, are due to the stimulation of catecholamine secretion, promoting energy expenditure and reducing the accumulation of body fat mass [168]. In the catechines class, green tea and coffee were also reported for the significant reduction of body weight and maintenance of the body weight in average condition. *Sorghum bicolor* L. has been demonstrated to be a worthy substitute to control obesity in weighty men because it reduces body fat fraction and amplifies daily carbohydrate and dietary fiber consumption when compared to wheat consumption [169]. Nowadays, people are having a great interest in herbal plant compositions or phytochemicals due to safer and efficacious effects, and the clinical studies must not be limited to *in vitro* or *in vivo* levels for their development.

## 8. Conclusions and Current and Future Inclinations on Biogenic Antiobesity Agents

Obesity is a multifaceted, long-lasting disorder triggered by an interaction of causative aspects such as lifestyle, dietary pattern, and environmental and genetic factors [140]. Healthy lifestyle and behavioral interventions are the main aspects of body weight loss and natural product-based treatment is more beneficial than synthesized molecules-based treatment in terms of toxicity [139–142]. Natural product-based molecules are also more beneficial in hyperlipidaemic activities, antidiabetic, cancer due to obesity, and hypercholesteremia [141, 142]. It is indeed that natural product-based molecules will provide the new and more appropriate platform for antiobesity treatment.

## Figures and Tables

**Table 1:** A comparison of FDA-approved antiobesity drugs [74].

Drug	Dose concentration	Approving bodies	Mechanism of action	Weight reduces up to kg/year	Side effects
Phentermine	46 mg–92 mg once daily.	Approved by FDA in 2012.	Reduces appetite	8.6	Dizziness, pulmonary hypertension
Lorcaserin	10 mg twice daily	Approved by FDA in 2012.	5-HT2C receptor activation	3.6	Headache, dizziness
Naltrexone	64 mg/720 mg tablets two times daily	Approved by FDA in 2014.	Noradrenaline and dopamine reuptake inhibitor.	4.8	Vomiting, dizziness
Orlistat	60–120 mg three times daily	Approved by FDA in 1999.	Pancreatic lipase inhibitor	3.4	Hepatotoxic, steatorrhea
Liraglutide	3.0 mg injection once daily	Approved by FDA in 2014.	GLP-1 receptor agonist	5.9	Nausea, pancreatitis

**Table 2:** Antiobesity phytochemicals' mechanism of action, signaling pathway, and their classification.

Mechanism of action	Signaling pathway	Classification	Phytochemical	Reference
Proliferation in energy outflow	(1) Browning of WAT into BAT	Flavanoid	Rutin, naringenin, luteolin, quercetin, genistein, and myricetin	[81–84]
	(2) Thermogenesis via UCP 1/UCP 3			
	(3) Escaping ATP formation	Phenols	p-hydroxybenzoic, cinnamic acid, ferulic acid, caffeic acid, p-coumaric, and cinnamic acid	[139–141]
	(4) PPAR-gamma, PGC 1 a, PRDM 16			

Craving suppressant influence	(1) Upregulation of POMC and CART pathway	Flavanoid	Apigenin, genistein, and cyanidin	[1–89]
	(2) Downregulation of AgRP and NPY pathway	Alkaloid	Halfordinol	[136–138]

Inhibition of lipase and other enzyme activity	(1) Inhibition of pancreatic phosphor lipase enzyme	Flavanoid	Epigallocatechin-3,5-digallate and other related flavan-3-ol-digallate esters, catechins, resveratrol, and galangin	[90–92]
	(2) Inhibition of alpha amylase enzyme	Anthocyanin	Cyanidin	

Adipocyte differentiation control	(1) Regulation of adipogenesis by PPAR-gamma, C/EBP families such as C/EBP alpha, beta, and epsilon	Flavanoid	Apigenin, guggul sterols, naringenin, genistein, hesperidin, myricetin, kaempferol, and rutin	[98–102]
	(2) The SREBP 1a, SREBP 1c, and SREBP 2 induce cholesterol biosynthesis. The SREBP 1c promotes differentiation of adipocytes and may activate PPAR-gamma. The inhibition of C/EBP alpha, PPAR-gamma, and SREBP may be effective for obesity treatment			
	(3) Adipocyte differentiation by apigenin is linked with inhibition of interleukin 6, leptin production, and monocyte chemoattractant protein 1(MCP-1). The suppression of the expression of PPAR-gamma, SREBP-1c, and GLUT-4 via JNK signaling	Alkaloid	Synephrine, nuciferine, piperine, and piperlongumine	
	(4) Interaction with PPAR- gamma and decreases adipocyte differentiation			

Regulation of fat metabolism activity	(1) Downregulation of perilipin-1	Flavanoid	Genistein, daidzein, kaempferol, apigenin, hesperidin, and berberine	[104, 105]
	(2) Promoting lipolysis and inhibition of insulin-dependent lipogenesis			
	(3) The downregulation of PPAR-gamma/EBP beta, SREBP-1, and genes of triglyceride biosynthesis	Phytosterols	Ampesterol, brassicasterol, guggulsterone, sitosterol, diosgenin, and stigmasterol	
	(4) The lowering of triacylglycerol concentrations in adipocytes by regulating lipolysis			

**Table 3:** Chemical structure and therapeutic application of phytochemicals in obesity.

Plant source	Name of phytochemical	Structure	Antiobesity effect	Phyto molecules	Reference
*Coriandrum sativum*	Quercetin	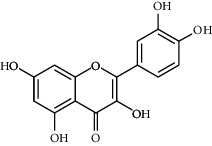	Reduces the process of adipogenesis by activation AMPK signaling mechanism	Flavonoids	[131]
*Curcuma longa*	Curcumin	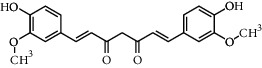	Enhanced *β*-oxidation, inhibition of fatty acid synthesis, and decreased fat storage	Flavonoids	[132]
*Vitis vinifera*	Catechins	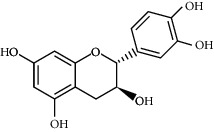	Prevents *α*-glucosidase activity and micelle formation in reducing carbohydrates absorption in the small intestine	Flavonoids	[133]
*Arachis hypogaea, Vitis vinifera, and Cyanococcus*	Resveratrol	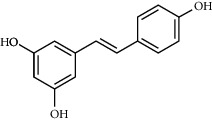	Prevents transcriptional activity and reduce adipogenesis	Flavonoids	[134]
*Alpinia galangal* and *Helichrysm aureonitens*	Galangin	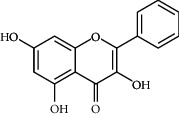	Reduces the collection of hepatic triglycerides	Flavonoids	[131–134]
*Cyclopia falcata* and *Cyclopia subternata*	Phloretin-3′,5′-di-c-glucoside	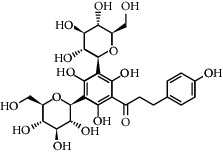	Inhibits the expression of peroxisome proliferator-activated receptor-2 (PPAR-2) and adipogenesis.	Flavonoids	[131–134]
*Glycine max*	Genistein	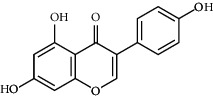	Antiadipogenic effects by suppressing PPAR-*α* and causing	Flavonoids	[131–134]
*Matricaria chamomilla*	Apigenin	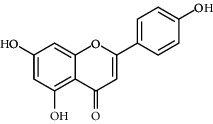	Antiobesity effect in visceral adipose tissue	Flavonoids	[136]
*Camellia sinensis and Coffea arabica*	Caffeine	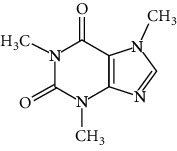	Exerts lipolytic and thermogenic actions	Alkaloids	[136]
*Capsicum annuum*	Capsaicin	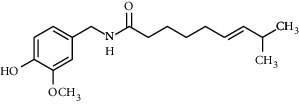	Enhanced lipid oxidation and increased energy expenditure	Alkaloids	[138]
*Nicotiana tabacum and Capsicum annum*	Nicotine	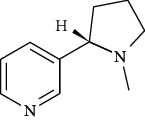	Prevents food intake and increase metabolic rate	Alkaloids	[136–138]
*Citrus aurantium*	p-synephrine	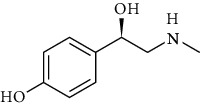	Increases metabolic rate and reduces weight loss	Alkaloids	[136–138]
*Aegle marmelos*	Halfordinol	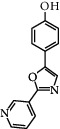	Prevents food intake and increase metabolic rate	Alkaloids	[136–138]
*Glycine max and Coffea canephora*	Chlorogenic acid	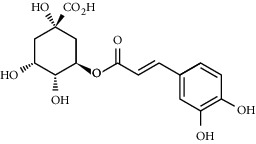	Reduces the absorption of carbohydrate	Phenols	[141]
*Hordeum vulgare and Asparagus officinalis*	Ferulic acid	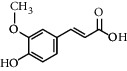	Improves the glucose and lipid homeostasis in a high-fat diet and reduce obesity	Phenols	[139]
*Coffea arabica*	Caffeic acid	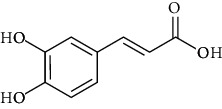	Modulated gut microbiota dysbiosis	Phenols	[140]
*Arachis hypogaea, Citrullus Colocynth, and Bauhinia variegate*	Sitosterol	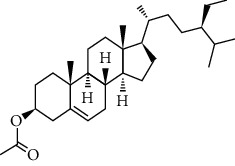	Reduce the absorption of cholesterol by lowering the level of cholesterol and LDL (low-density lipoprotein)	Phytosterol	[142]
*Dioscorea villosa*	*β*-sitosterol	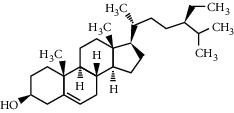	Exhibited antiobesity effects by suppressing sterol regulatory elements	Phytosterol	[143]
*Trigonella foenumgraecum* and *Dioscorea villosa*	Diosgenin	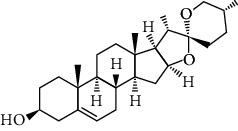	Inhibits accumulation of triglyceride and expression of lipogenic genes	Phytosterol	[144]
*Trapa natans* and *Tribulus terrestris*	Protodioscin	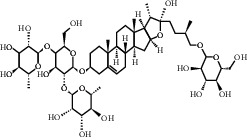	Reduces blood levels of triglyceride, cholesterol, LDL	Phytosterol	[142–144]
*Punica granatum* and *Momordica*	Punicic acid	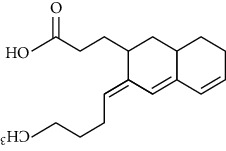	Enhances the activity of PPAR-*α*, PAR *γ*-responsive genes and reduces the deposition of adipose tissue	Terpenoid	[145–148]
*Orthosiphon aristatus*	Betulinic acid	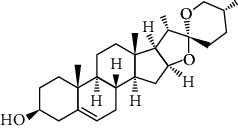	Suppresses tyrosine phosphatase 1B and enhances the antiobesity potential	Terpenoid	[145–148]

**Table 4:** Efficacy of natural products-based antiobesity bioactive components.

S. N.	Name of the natural product	Bioactive component	Duration of HFD (high-fat diet) in male mice	Reduction in body weight in (%)	References
1	*Morus alba*	Resveratrol anthocyanin	HFD (fat: 45%, w/w) for 12 weeks	53.5%	[151]
2	*Curcuma longa*	Curcumin	HFD (fat: 60%, w/w) for 12 weeks	15.9%	[152]
3	*Rhizoma coptidis*	Berberine	HFD (fat: 16.2%, w/w) for 6 weeks	13.2%	[153]
4	*Capsicum annuum*	Capsicin	HFD (fat: 45%, w/w) for 9 weeks	8%	[154]
5	*Acacia mollissima*	Robinetinidol	HFD (fat: 60%, w/w) for 7 weeks	23.2%	[155]
6	*Zingiber officinale*	Gingerol, paradol, d shogoal	HFD (fat: 30%, w/w) for 5 weeks	38.6%	[156]
7	*Nelumbo nucifera*	Alkaloids	HFD (fat: 20%, w/w) for 6 weeks	9.81%	[157]
8	*Camellia sinensis*	Caffeine	HFD (10%, w/w) for 6 weeks	11.3%–16.9%;	[107]
9	*Coffea Arabica*	Caffeoyl, quinic acids	HFD (fat: 30%, w/w) for 2–15 weeks	14.3%	[158]
10	*Glycine max*	Protein isolated	HFD (25%, w/w) for 12 weeks	10.0%.	[159]
11	*Vaccinium ashei*	Anthocyanins	HFD (45%, w/w) for 12 weeks	9.81%	[160]
12	*Citrus depressa*	Flavonoids	HFD (35%, w/w) for 4 weeks	10.0%.	[161]

## Data Availability

All the key information is already available in the manuscript. Still, the authors are ready to share the raw data if the proper channel for the inquiry will be followed which will be routed through journal and affiliation authorities.

## Abbreviations

JAK: Janus kinase
STAT: Signal transducer and activator of transcription
SH2: Src homology 2
SOCS: Suppression of cytokine signaling
PTP: Protein tyrosine phosphatase
POMC: Proopiomelanocortin
NPY: Neuropeptide Y
CART: Cocaine- and amphetamine-related transcript
MSH: Melanocyte stimulating hormone
AgRP: Agouti-related protein
MC4R: Melanocortin 4 receptor
CGRP: Calcitonin gene-related protein
BAT: Brown adipose tissue
WAT: White adipose tissue
GWAS: Genome-wide association studies
FTO: Fat mass and obesity
EDC: Endocrine disrupting chemicals
C/EBP: CCAAT enhancer binding protein
PPAR: Peroxisome proliferator-activated receptor
US-FDA: U.S. Food and Drug Administration
UCP: Uncoupling protein
SREBP: Sterol regulatory element binding protein
MUFA: Monounsaturated fatty acid
PUFA: Polyunsaturated fatty acid
EPA: Eicosapentaenoic acid
DHA: Docosahexaenoic acid
DPA: Docosapentaenoic acid
DF: Dietary fibers
SDF: Soluble dietary fiber
IDF: Insoluble dietary fiber
SCFAs: Short-chain fatty acids
HPD: High-protein diet.
